# Enzymatic Valorisation of Hazelnut Skin and Hemp Shives By-Products

**DOI:** 10.3390/ma19214589

**Published:** 2026-10-29

**Authors:** Ester De Martino, Angela Sorrentino, Alfredo Sánchez, Reynaldo Villalonga, Aniello Falciano, Carmine Buonaiuto, Prospero Di Pierro

**Affiliations:** 1 Department of Agricultural Sciences, University of Naples Federico II, Piazza Carlo di Borbone 1, 80055 Portici, Naples, Italyprospero.dipierro@unina.it (P.D.P.); 2 Centre for Innovation and Development in Food Industry (CAISIAL), University of Naples Federico II, Via Università 133, 80055 Portici, Naples, Italy; 3 Nanosensors and Nanomachines Group, Department of Analytical Chemistry, Faculty of Chemistry, Complutense University of Madrid, Plaza de las Ciencias 2, Ciudad Universitaria, 28040 Madrid, Spainrvillalonga@quim.ucm.es (R.V.)

**Keywords:** hazelnut skin, hemp shives, enzymatic hydrolysis, by-products, lignocellulosic materials

## Abstract

**Highlights:**

- Enzymatic treatments differently affected hazelnut skins and hemp shives.
- Hemicellulase induced the most pronounced matrix structural changes.
- Enzymatic treatment increased phenolics and antioxidant activity in liquid fractions.
- Enzymatic conversion can tailor the properties of lignocellulosic residues.
- Structural changes were associated with altered functional properties.
- Enzyme-assisted processing supports integrated biomass valorisation.

**Abstract:**

The valorisation of lignocellulosic agro-industrial residues through mild and sustainable processes represents a promising strategy for their conversion into value-added fractions. This study investigated an enzyme-assisted approach for the valorisation of hazelnut skins (HaSk) and hemp shives (HeSh), evaluating the effects of cellulase, hemicellulase, pectinase and xylanase treatments on sugar release, structural and morphological characteristics, functional properties of the residual solids, and phenolic content and antioxidant activity of the liquid fractions. Enzymatic treatments produced substrate- and enzyme-dependent effects. Hemicellulase showed the most pronounced general effects, particularly in terms of sugar release and modification of the solid matrices. Scanning electron microscopy confirmed changes in the surface morphology of treated materials. Enzymatic treatment also differently affected the functional properties of the two substrates, with HaSk showing enzyme-dependent changes in WHC, whereas HeSh maintained comparable or increased hydration properties and showed increased OHC following hemicellulase treatment. In the liquid fractions, enzymatic treatment increased total phenolic content and antioxidant activity, indicating enhanced release of phenolic compounds from the lignocellulosic matrices. Overall, the results support enzyme-assisted processing as a mild strategy for the valorisation of heterogeneous lignocellulosic residues, generating solid fractions with modified functional properties and liquid fractions enriched in phenolic compounds.

## 1. Introduction

The transition towards a circular bioeconomy has stimulated growing interest in the sustainable valorisation of agro-industrial residues as renewable feedstocks for the production of bio-based materials, chemicals and functional ingredients. The European Union alone generates approximately 956 million tonnes of agricultural biomass annually, nearly half of which consists of crop residues, highlighting the enormous potential of these underutilised resources for the development of sustainable biorefinery processes [1]. Furthermore, agricultural residues from the main European crops were estimated to account for approximately 419 million tonnes of dry lignocellulosic biomass per year in the EU28, representing a major renewable carbon resource for non-food applications [2]. More recent studies have further highlighted the potential of agricultural lignocellulosic residues as feedstocks for integrated valorisation strategies and the development of biofuels, biomaterials and other value-added products [3,4].

Despite their abundance, many of these by-products remain underexploited or are directed towards low-value applications, although representing valuable sources of structural polysaccharides and bioactive compounds [4]. In recent years, the biorefinery concept has emerged as a promising strategy to maximise resource efficiency by converting biomass into multiple value-added products while minimising waste generation. Among the different renewable feedstocks, lignocellulosic agro-industrial residues constitute an attractive source of carbohydrates, phenolic compounds and structural polymers for the development of sustainable bioprocesses and functional materials [5,6].

Lignocellulosic biomass is a highly heterogeneous composite material mainly composed of cellulose, hemicellulose and lignin, with minor amounts of pectin, proteins, extractives and mineral components. These polymers are not randomly distributed but are intricately assembled into a hierarchical three-dimensional architecture, where cellulose microfibrils are embedded within a matrix of hemicelluloses and lignin [7]. Furthermore, covalent linkages between lignin and carbohydrates give rise to lignin–carbohydrate complexes (LCCs) [8], while the complex architecture and interactions within the lignin–carbohydrate matrix contribute to biomass recalcitrance by limiting enzyme accessibility to structural polysaccharides [9]. Consequently, the efficient fractionation and utilisation of lignocellulosic biomass remain major technological challenges and generally require pretreatment strategies capable of increasing the accessibility of carbohydrate polymers while preserving their potential for subsequent valorisation.

Among the available valorisation strategies, enzyme-assisted processing has attracted considerable attention because it enables the selective hydrolysis of lignocellulosic polymers under mild operating conditions, thereby reducing energy consumption and the use of harsh chemicals compared with conventional extraction technologies [10,11,12]. Owing to their high substrate specificity, carbohydrolases can selectively target different structural components of the plant cell wall, promoting the disassembly of the lignocellulosic network and facilitating the release of soluble compounds [4,12,13]. In particular, cellulases, hemicellulases, pectinases and xylanases have been widely investigated for the recovery of fermentable sugars, oligosaccharides and phenolic compounds from plant-derived biomasses, while simultaneously modifying the physicochemical properties of the residual solid fraction [9,14,15]. Nevertheless, the effectiveness of enzymatic hydrolysis strongly depends on the structural organisation and chemical composition of the substrate, making the selection of appropriate enzymatic preparations and pretreatment strategies a crucial step for biomass valorisation.

Hazelnut skins (HaSk) and hemp shives (HeSh) are two representative agro-industrial lignocellulosic residues that remain largely underutilised despite their considerable potential as renewable feedstocks for biorefinery applications. Hazelnut skins, the thin perisperm surrounding the kernel, are generated during the roasting process and are particularly rich in dietary fibre and phenolic compounds, making them an attractive source of both structural polysaccharides and antioxidant phytochemicals [16,17,18]. In contrast, hemp shives, the woody core remaining after fibre extraction from hemp stems, are characterised by a highly lignocellulosic structure with considerable potential for the production of bio-based materials and chemicals within integrated biorefinery schemes [19,20,21]. Despite their different composition and potential applications, comparative studies evaluating the response of these two biomasses to enzyme-assisted processing remain limited.

Although enzyme-assisted processing has emerged as a promising strategy for the sustainable valorisation of lignocellulosic biomass [5,6,22], the response of different agro-industrial residues to enzymatic treatment remains highly substrate-dependent. Since the chemical composition and structural organisation of lignocellulosic matrices vary considerably according to their botanical origin, comparative investigations are needed to better understand the effectiveness of different carbohydrolases and to support the selection of suitable enzymatic strategies for specific biomasses [8]. Such studies are essential for the development of efficient and sustainable biomass valorisation processes within integrated biorefinery and multi-valorisation approaches [22].

Based on these considerations, the aim of the present work was to evaluate enzyme-assisted processing as a process with potential environmental advantages for the valorisation of two agro-industrial lignocellulosic by-products, hazelnut skins (HaSk) and hemp shives (HeSh), characterised by markedly different chemical composition. Different carbohydrolases were selected according to the polymeric composition of each biomass, and their effectiveness was assessed through the determination of soluble reducing and total sugars released into the liquid fraction, together with the evaluation of structural, chemical and functional modifications occurring in the residual solid fraction. In addition, the recovery of phenolic compounds and the antioxidant activity of the liquid hydrolysates were investigated to provide a broader assessment of the potential of enzymatic processing for biomass valorisation.

## 2. Materials and Methods

### 2.1. Enzymes, Chemicals and Raw Materials

The commercial enzyme preparations were purchased from Sigma Aldrich (cellulase and hemicellulase, Merck KGaA, Darmstadt, Germany), Sunson Enzyme (pectinase, Beijing, China) and Creative Enzymes (xylanase, Shirley, NY, USA). Cellulase, hemicellulase and pectinase were from Aspergillus niger, while xylanase was from Bacillus subtilis. Phenol, Gallic acid and 2,2-diphenyl-1-picrylhydrazyl (DPPH) were from Merck Life Science S.r.l., Milan, Italy. Folin–Ciocalteau reagent, 2,4-dinitrosalicylic acid (DNS), Acetone, Ethanol and all other chemical reagents used for the experiments were of analytical grade and purchased by Carlo Erba, Milan, Italy. Hazelnut skins (*Corylus avellana* L., HaSk) were kindly provided by EuroNut SpA (San Paolo Bel Sito, Italy), while hemp shives (*Cannabis sativa* L., HeSh) were supplied by Deltafina S.r.l. (Orvieto, Italy).

### 2.2. Pre-Treatments, Proximate and Fibre Composition

The raw materials, namely hazelnut skins (HaSk) and hemp shives (HeSh), were micronized using a laboratory hammer mill (LM 3100, Perten, Rozzano, Italy) equipped with a 0.5 mm cut-off grid. The particle size distribution is reported in Figure S1 (Supplementary Materials). The proximate composition of the micronized powders was determined according to AOAC International [23]: Lipids were extracted by Soxhlet extraction using hexane as solvent; crude protein content was determined by the Kjeldahl method using a nitrogen-to-protein conversion factor of 6.25; ash content was measured by incineration at 575 °C in a muffle furnace.

The polymeric composition of micronized HaSk and HeSh was determined according to the protocol described by Ayeni et al. [24] with minor modifications. After Soxhlet, the defatted samples were dried to constant weight in a convection oven at 105 °C. Subsequently, the dried biomass (1 g) was suspended in 150 mL of 0.5 M NaOH and heated at 100 °C for 3 h. After cooling to room temperature, the solid fraction was recovered by vacuum filtration and then washed with distilled water until neutral pH. The residue was then dried to constant weight at 105 °C.

Hemicellulose content was calculated gravimetrically as the difference between the sample weight before and after alkaline hydrolysis and expressed as % (*w*/*w*) on a dry matter basis after correction for protein and ash contents.

For lignin estimation, 0.3 g of dried defatted biomass were treated with 3 mL of 72% (*w*/*w*) H_2_SO_4_ at room temperature for 2 h with gentle stirring every 30 min; subsequently, 84 mL of distilled water were added and the hydrolysis was completed in an autoclave at 121 °C for 1 h. After cooling to room temperature, the slurry was vacuum filtered through a filtering crucible.

Acid-insoluble lignin was determined gravimetrically after drying the residue at 105 °C and correcting for ash content by incineration at 575 °C in a muffle furnace. Acid-soluble lignin fraction was determined spectrophotometrically by measuring the absorbance of the filtrate at 320 nm. Total lignin content was calculated as the sum of acid-insoluble and acid-soluble lignin fractions. Finally, cellulose content was calculated by difference, assuming that cellulose, hemicellulose, lignin, proteins, extractives and ash accounted for the total biomass composition.

### 2.3. Enzymatic Treatment

The enzymatic activity of each preparation was experimentally determined under its respective optimal pH and temperature conditions using the corresponding substrate: low-methoxyl pectin for pectinase, cellulose for cellulase, arabinoxylan for hemicellulase, and xylan for xylanase. Activity was quantified based on the release of reducing sugars using the DNS method described below. One unit (U) of enzymatic activity was defined as the amount of enzyme releasing 1 µmol of reducing sugars per minute under the assay conditions. The amount of each enzyme preparation added to the biomass was calculated to provide a nominal activity of 150 U/g of dry biomass. Since the activity assays employed different substrates and enzyme-specific conditions, the resulting activities provide a standardized basis for dosage within each assay but do not imply equivalent catalytic efficiencies on the raw biomass across the different enzyme preparations.

Thus, the previously micronized and thermally treated materials were subjected to enzymatic hydrolysis using 150 U of enzyme per gram of sample under continuous agitation at 180 rpm in an orbital shaker (Forma Scientific, Marietta, OH, USA). Assay parameters were evaluated and standardized as follows: incubation times ranged from 3 to 18 h; enzyme-specific optimal temperatures were set at 37 °C, 40 °C, 45 °C and 65 °C for cellulase, hemicellulase, pectinase and xylanase, respectively; pH conditions were selected according to manufacturer’s guidelines, using acetate buffer at pH 5, 4.5 and 4 for cellulase, hemicellulase and pectinase, respectively, and phosphate buffer at pH 6 for xylanase. At the end of the incubation period, enzymatic activity was inactivated by heating the samples in a water bath at 100 °C for 10 min. Samples were then centrifuged at 14,000× *g* for 10 min to separate the supernatant and pellet fractions. Both fractions were collected and subjected to subsequent analyses.

### 2.4. Reducing and Total Sugars Determination

The determination of reducing sugars released after enzymatic treatment was performed according to the protocol described by Miller [25]. Briefly, 0.5 mL of DNS reagent were added to 0.5 mL of sample aliquot; when necessary, samples were appropriately diluted to ensure that sugar concentrations fell within the calibration range. The mixtures were heated in a boiling water bath for 5 min and subsequently cooled to room temperature under running tap water before absorbance measurement. Then, 5 mL of distilled water were added, and absorbance was measured at 540 nm using a UV–VIS spectrophotometer (V-730, JASCO International Co. Ltd., Sennincho Hachioji, Japan).

The reducing sugar content was quantified based on the development of the DNS chromogenic reaction, whose intensity was proportional to the concentration of reducing sugars. Quantification was performed using a glucose calibration curve ranging from 10 to 200 µg/mL (y = 0.0052x + 0.0128; R^2^ = 0.9984), and results were expressed as glucose equivalents.

Total sugars were determined according to the method described by Dubois et al. [26]. Briefly, 0.4 mL of sample solution, appropriately diluted with distilled water according to sugar concentration, was mixed with 0.2 mL of 5% (*w*/*v*) phenol solution and 1 mL of 98% sulfuric acid. After cooling, colour intensity was measured at 490 nm using a UV–VIS spectrophotometer. A glucose calibration curve in the range of 5–40 µg/mL, with the regression equation y = 0.0649x + 0.0261 (R^2^ = 0.9993), was used to estimate the glucose equivalents concentration in the samples.

### 2.5. SEM Analysis

Morphological changes induced by enzymatic treatment were evaluated by scanning electron microscopy (SEM) by comparing treated samples with their corresponding untreated controls. Prior to observation, samples were mounted on aluminum stubs and gold-coated by DC sputtering to improve electrical conductivity. Microstructural observations were performed at magnifications ranging from 500× to 2000× using a scanning electron microscope (JSM-7600F, JEOL Ltd., Tokyo, Japan) operated at an accelerating voltage of 20 kV. Representative micrographs were acquired for all samples and used for morphological evaluation.

### 2.6. ATR-FTIR Analysis

ATR-FTIR spectra of lyophilized samples were recorded using a Bruker Alpha spectrometer (Bruker Optics, Billerica, MA, USA) equipped with a diamond attenuated total reflectance (ATR) crystal. Spectra were collected at room temperature over the spectral range of 400–4000 cm^−1^. A pressure clamp was used to ensure optimal contact between the powdered samples and the ATR crystal, providing consistent sample thickness and improving measurement reproducibility. Data acquisition and spectral processing were performed using OPUS software (Version 7.5.18, Bruker GmbH, Ettlingen, Germany). Spectra were acquired at a resolution of 2 cm^−1^ by averaging 40 scans for each measurement. Three independent spectra were acquired for each sample.

### 2.7. Water- and Oil-Holding Capacity (WHC and OHC)

Water-holding capacity (WHC) and oil-holding capacity (OHC) were determined to assess the functional properties of the lignocellulosic residues before and after enzymatic treatment. These parameters were selected to investigate whether enzyme-induced modifications of the biomass structure affected its ability to retain water and oil, providing complementary information to the evaluation of soluble sugar release and supporting the assessment of the potential functional applications of the residual materials.

For the determination of the water-holding capacity (WHC), 1 g of dried lignocellulosic sample was mixed with 10 mL of distilled water, vortexed for 1 min, and incubated at room temperature in an orbital shaker (Forma Scientific) at 150 rpm for 30 min. The suspensions were then centrifuged at 4000× *g* for 30 min at room temperature to separate the pellets from the supernatants. After centrifugation, the tubes were drained at a 45° angle for 5 min before being weighed. WHC was expressed as grams of water retained per gram of dry sample and calculated using Equation (1), adapted from Robertson and Eastwood [27]:

(1) $$\mathrm{WHC}=\;\frac{m_{\mathrm{wet}}\;-\;m_{\mathrm{dry}}\;}{m_{\mathrm{dry}}}$$

where *m_wet_* is the mass of the hydrated sample after centrifugation and removal of the supernatant, and *m_dry_* is the initial dry sample mass.

The oil-holding capacity (OHC) was determined following the same procedure, except that distilled water was replaced with 15 mL of sunflower oil. The suspension was vortexed for 1 min every 5 min during a total incubation period of 30 min. The resulting suspension was centrifuged at 3000× *g* for 30 min, after which the unbound oil was carefully decanted and discarded and residual oil adhering to the tube walls was removed using absorbent paper. The mass of the oil-containing pellet was then used to calculate OHC using Equation (2), adapted from Robertson and Eastwood [27]:

(2) $$\mathrm{OHC}=\;\frac{m_{\mathrm{oil}}\;-\;m_{\mathrm{dry}}\;}{m_{\mathrm{dry}}}$$

where *m_oil_* is the mass of oil-containing sample after centrifugation and removal of unbound oil, and *m_dry_* is the initial dry mass.

All WHC and OHC measurements were performed in triplicate.

### 2.8. Total Phenolic Content (TPC) and DPPH Radical Scavenging Activity

The total phenolic content (TPC) was determined according to the method described by Sun et al. [28], with slight modifications. Briefly, 50 µL of sample extract was mixed with 70 µL of Folin–Ciocalteu reagent and 880 µL of distilled water, vortexed for 1 min, and incubated at room temperature for 5 min. Subsequently, 530 µL of distilled water and 70 µL of 7.5% (*w*/*v*) sodium carbonate solution were added, and the mixtures were incubated at 45 °C in the dark for 15 min. Absorbance was measured at 760 nm using a UV–VIS spectrophotometer (V-730, JASCO International Co. Ltd., Sennincho Hachioji, Japan). Gallic acid was used as the calibration standard, and the results were expressed as milligrams of gallic acid equivalents (GAEs) per gram of dry weight (d.w.). Quantification was performed using a gallic acid calibration curve ranging from 1 to 20 µg/mL (y = 0.0674x − 0.0205; R^2^ = 0.9992).

The antioxidant activity of the extracts was evaluated using the DPPH radical scavenging assay according to Duan et al. [29]. Briefly, 950 µL of freshly prepared 0.1 mM DPPH methanolic solution was mixed with 50 µL of sample extract or methanol (blank). The reaction mixtures were incubated in the dark at room temperature for 1 h, after which absorbance was measured at 517 nm using the same UV–VIS spectrophotometer. For accurate analysis, the absorbance of the sample (50 µL) simply diluted in methanol (950 µL) was also measured. The DPPH radical scavenging activity was calculated according to Equation (3):

(3) $$\mathrm{DPPH}\;\mathrm{scavenging}\;\mathrm{activity}\;\left(\%\right)=\;\frac{A_{b}-{\;A}_{s}-A_{0}}{A_{b}}\;\times 100$$

where *A_b_* is the absorbance of the blank sample, *A_s_* and *A_0_* are the absorbances of the sample with and without DPPH solution, respectively.

### 2.9. Statistical Analysis

All experiments and analytical measurements were performed in three independent replicates, and the results are presented as the mean ± standard deviation (SD). Statistical analyses were performed using XLSTAT software (Version 2023.11.08, Addinsoft, Paris, France). Data were analysed by one-way analysis of variance (ANOVA), followed by Tukey’s post hoc test to assess statistically significant differences among all experimental values included in each graph. Differences were considered statistically significant at *p* < 0.05.

### 2.10. Generative AI Disclosure

During the preparation of this manuscript, the authors used ChatGPT (OpenAI, ver GPT-4o) to assist with English language editing, to improve the clarity and readability of the text, and support the identification, organization, and formatting of bibliographic references. ChatGPT was also used to assist in generating the graphical abstract. The authors critically reviewed and revised all AI-assisted outputs and take full responsibility for the final content of the manuscript, including the graphical abstract. No AI tools were used to generate or analyze the experimental data, perform statistical analyses, or draw scientific conclusions.

## 3. Results and Discussion

### 3.1. Physicochemical Characterization of the Raw Biomasses

The physicochemical characterization revealed marked differences in the composition of hazelnut skins (HaSk) and hemp shives (HeSh), particularly in the relative abundance of cellulose, hemicellulose and lignin, despite both biomasses exhibiting the typical characteristics of lignocellulosic materials (Table 1). The structural organization of these polymers within the plant cell wall plays a key role in determining biomass recalcitrance by limiting enzyme accessibility to structural carbohydrates through the formation of a complex lignin–carbohydrate network [8]. Therefore, differences in the relative abundance of these structural components are expected to influence the efficiency of enzymatic hydrolysis.

HaSk was characterized by a higher cellulose content (45.15 ± 0.13% d.w.) and a lower proportion of hemicellulose (14.85 ± 0.65% d.w.) than HeSh, which exhibited a more balanced lignocellulosic composition, with cellulose and hemicellulose accounting for 37.11 ± 2.14% and 25.12 ± 2.12% d.w., respectively. Lignin content was comparable between the two biomasses (29.58 ± 4.07% for HaSk and 27.59 ± 3.15% for HeSh), whereas only minor differences were observed in protein, ash, and hexane extractives. In general, these findings indicate that HaSk is characterized by a cellulose-rich structure, whereas HeSh contains a relatively higher proportion of hemicellulosic polysaccharides, suggesting that the accessibility of structural polysaccharides, rather than their abundance alone, is likely to govern the effectiveness of the different carbohydrolases.

The composition determined for HaSk was generally consistent with previous studies, which identified cellulose as the predominant structural polysaccharide together with lower hemicellulose content, although slight variations have been reported depending on cultivar, roasting conditions and analytical methodology [17,30,31]. Likewise, the composition of HeSh agreed with values reported in the literature, while confirming the well-known variability associated with hemp cultivar, geographical origin, harvesting stage and biomass processing [19].

Based on these compositional differences, HaSk and HeSh were expected to exhibit different susceptibilities to carbohydrolase-assisted hydrolysis, reflecting both enzyme substrate specificity and the accessibility of structural polysaccharides within each biomass. This hypothesis was investigated in the following sections by evaluating the release of soluble sugars together with the structural, morphological and functional modifications induced by the different enzymatic treatments.

### 3.2. Pretreatment Selection and Evaluation of Enzymatic Hydrolysis Through Soluble Sugar Release

The distinct lignocellulosic composition of hazelnut skins (HaSk) and hemp shives (HeSh), highlighted in Section 3.1, suggested that the efficiency of enzymatic hydrolysis would strongly depend on substrate accessibility. Since the compact organization of cellulose, hemicellulose and lignin limits enzyme penetration, an appropriate pretreatment is generally required to partially disrupt the lignocellulosic network and improve enzyme accessibility to structural polysaccharides [32].

Preliminary physical and chemical pretreatments were therefore evaluated to reduce particle size and remove soluble compounds from the biomass before enzymatic hydrolysis (Figures S1 and S2), thus enhancing biomass accessibility to enzymes. Micronization effectively reduced particle size, while different washing procedures were compared by measuring the total sugar content of the resulting supernatants. As shown in Figure S2, treatment with water at 100 °C for 10 min resulted in the highest release of total sugars among the tested washing conditions, including ethanol, acetone, and water at room temperature. This condition was therefore selected for subsequent enzymatic hydrolysis experiments. The choice also avoided the use of organic solvents; however, the environmental sustainability of the overall process was not directly evaluated.

**Figure 1:**
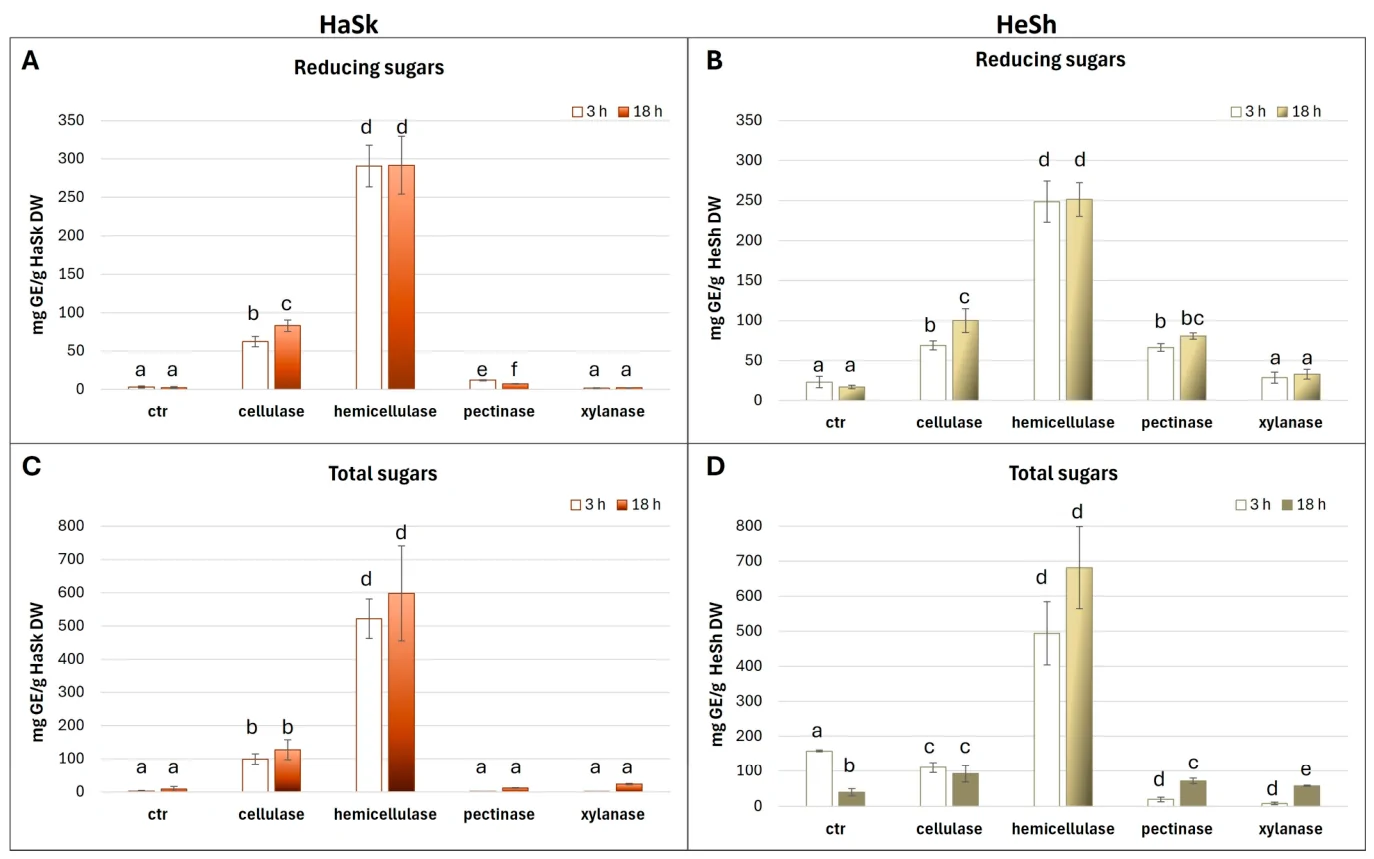
Evaluation of enzymatic activity of carbohydrolases on hazelnut skins (HaSk, (**A**,**C**)) and hemp shives (HeSh, (**B**,**D**)) through analysis of soluble reducing and total sugars released in the liquid fraction. GE, glucose equivalents; ctr, control without enzymes. Data expressed as mean ± SD of three replicates (n = 3). Different letters indicate significant differences (*p* < 0.05).

The efficiency of enzymatic hydrolysis was assessed by quantifying both reducing sugars (DNS assay) and total soluble sugars (phenol–sulfuric acid assay) released into the liquid fraction (Figure 1). In particular, hemicellulase exhibited the highest hydrolytic efficiency for both biomasses, whereas cellulase, pectinase and xylanase produced significantly lower sugar yields.

Regarding reducing sugars (Figure 1A,B), hemicellulase rapidly reached its maximum hydrolytic activity. In HaSk, glucose equivalents reached 290.63 mg g^−1^ after 3 h of treatment and remained essentially unchanged after 18 h (291.69 mg g^−1^).

A similar behaviour was observed for HeSh, where glucose equivalents increased only marginally from 248.58 to 251.36 mg g^−1^ between 3 and 18 h. These results indicate that the hydrolysis of the most accessible polysaccharidic regions occurred rapidly during the initial stages of incubation, whereas extending the reaction time did not result in a substantial increase in reducing sugar release.

To gain further insight into the evolution of the hydrolytic process, total soluble sugars were quantified using the phenol–sulfuric acid assay (Figure 1C,D). In contrast to reducing sugars, total sugar concentration continued to increase during prolonged incubation. In HaSk, glucose equivalents increased from 521.70 mg g^−1^ after 3 h to 598.05 mg g^−1^ after 18 h, while in HeSh, they increased from 494.21 to 681.61 mg g^−1^ over the same incubation period. This behaviour suggests that prolonged enzymatic treatment progressively disrupted the lignocellulosic architecture, favouring the solubilization of carbohydrate fractions that were initially embedded within the biomass matrix. Consequently, although the release of reducing sugars reached a plateau after the initial hydrolysis phase, the continued structural disassembly of the cell wall likely promoted the extraction of soluble oligosaccharides and larger carbohydrate fragments, resulting in a progressive increase in total sugar content.

A similar progressive release of soluble carbohydrates during prolonged enzymatic hydrolysis of lignocellulosic biomasses has been reported by Covino et al. [33]. Among the tested enzymes, hemicellulase consistently provided the highest sugar yields for both biomasses. This superior performance can be attributed not only to the heterogeneous and highly branched structure of hemicellulose but also to the nature of hemicellulase preparations, which generally consist of a complex enzymatic cocktail including xylanases, mannanases and several accessory glycoside hydrolases acting synergistically on different hemicellulosic domains [34]. The coordinated action of these enzymes facilitates the cleavage of both backbone and side-chain linkages, increasing substrate accessibility and promoting the release of soluble carbohydrates [35,36].

The hydrolysis yields obtained in the present study can also be considered in relation to previous investigations on the enzymatic conversion of lignocellulosic biomasses. Marcolongo et al. [37] investigated the enzymatic hydrolysis of hemp biomass following steam-explosion or alkaline pretreatment and reported different sugar yields depending on the pretreatment applied. Other studies on industrial hemp have likewise highlighted the importance of pretreatment conditions and substrate accessibility in determining enzymatic digestibility and sugar recovery [38]. These findings support the role of the physicochemical characteristics of the biomass in determining hydrolysis efficiency. Nevertheless, direct quantitative comparisons with the present results are limited by differences in biomass fractions, pretreatment procedures, enzyme systems and analytical methods. In the present study, hemicellulase treatment resulted in substantial soluble sugar release from both biomasses, without the need for an intensive chemical pretreatment.

**Figure 2:**
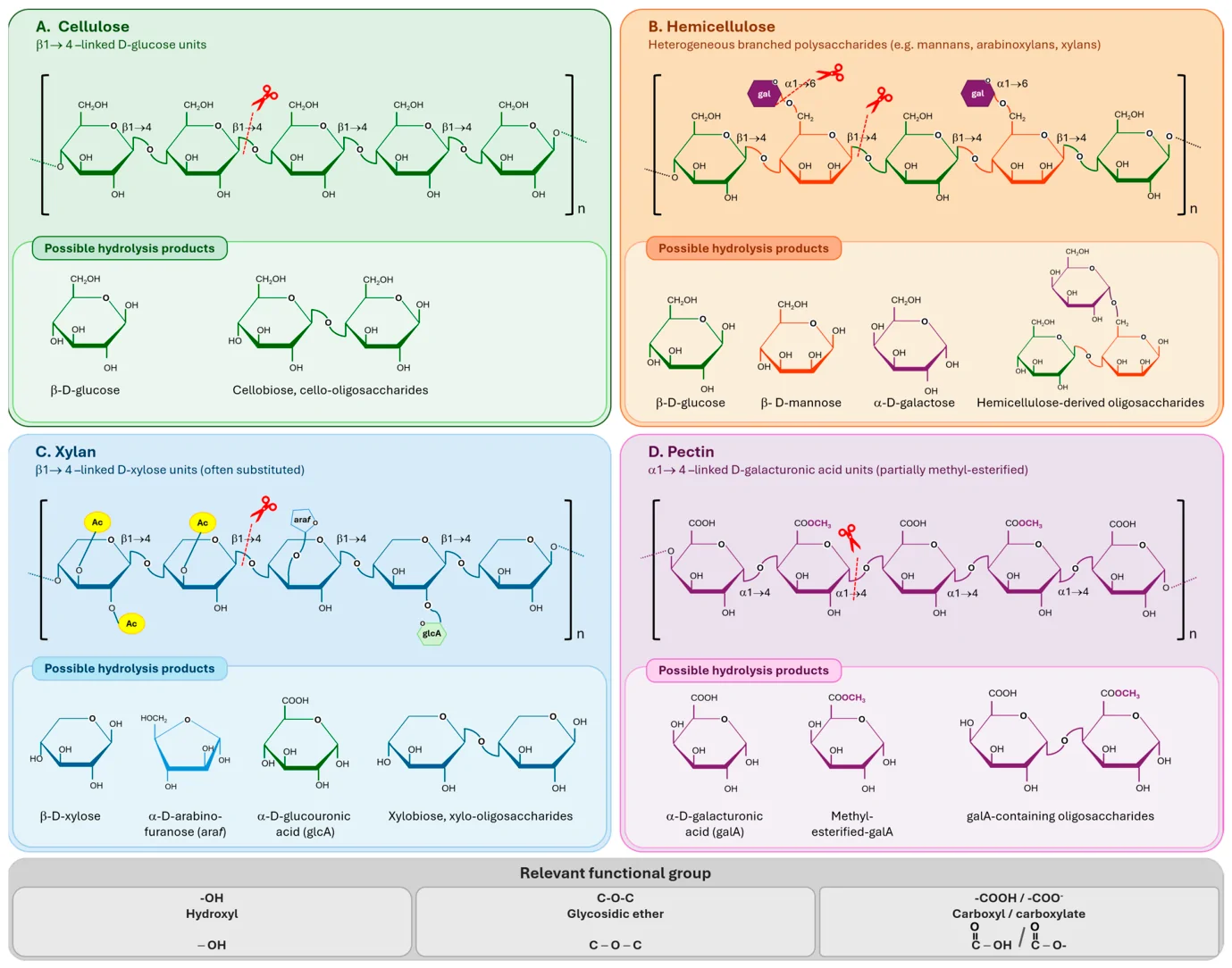
Schematic representation of plausible products of enzymatic hydrolysis of cellulose (**A**), hemicellulose (**B**), xylan (**C**) and pectic polysaccharides (**D**), highlighting the main functional groups associated with carbohydrate structures (grey box at the bottom). The scissors indicate the likely cutting sites for the enzymes. The proposed products are hypothetical and are intended to illustrate possible hydrolysis pathways; individual sugars and oligosaccharides were not identified experimentally in this study.

For hazelnut skin, previous research has also investigated the nature of its soluble carbohydrate fractions. Montella et al. [39] identified water- and alkali-soluble oligosaccharides in hazelnut skin, including galacturonic acid-containing oligosaccharides and xyloglucan-related structures. Although these findings cannot be directly compared with the enzymatic hydrolysis yields obtained in the present study, they demonstrate the presence of structurally diverse carbohydrate fractions in this biomass. The substantial sugar release observed following hemicellulase treatment suggests that this enzyme preparation promoted the solubilization of carbohydrate components in HaSk. However, the specific polysaccharides hydrolysed cannot be identified from the DNS and phenol–sulfuric acid assays alone. For illustrative purposes, Figure 2 provides a schematic representation of plausible products arising from the hydrolysis of cellulose, hemicellulose, xylan and pectic polysaccharides, highlighting the main functional groups associated with these carbohydrate structures. These representations are hypothetical and do not imply the experimental identification of specific sugars or oligosaccharides.

Interestingly, despite the higher hemicellulose content of HeSh compared with HaSk (Table 1), hemicellulase proved to be the most effective enzyme for both biomasses, indicating that hydrolysis efficiency depends not only on substrate abundance but also on the accessibility of structural polysaccharides and the synergistic action of multiple enzymatic activities.

The higher hydrolytic efficiency achieved by hemicellulase suggests that significant structural modifications occurred within both lignocellulosic matrices during enzymatic treatment. To verify this hypothesis, the morphological and structural changes induced by enzymatic hydrolysis were further investigated by scanning electron microscopy (SEM) and ATR-FTIR spectroscopy.

### 3.3. Morphological Changes Induced by Enzymatic Hydrolysis

**Figure 3:**
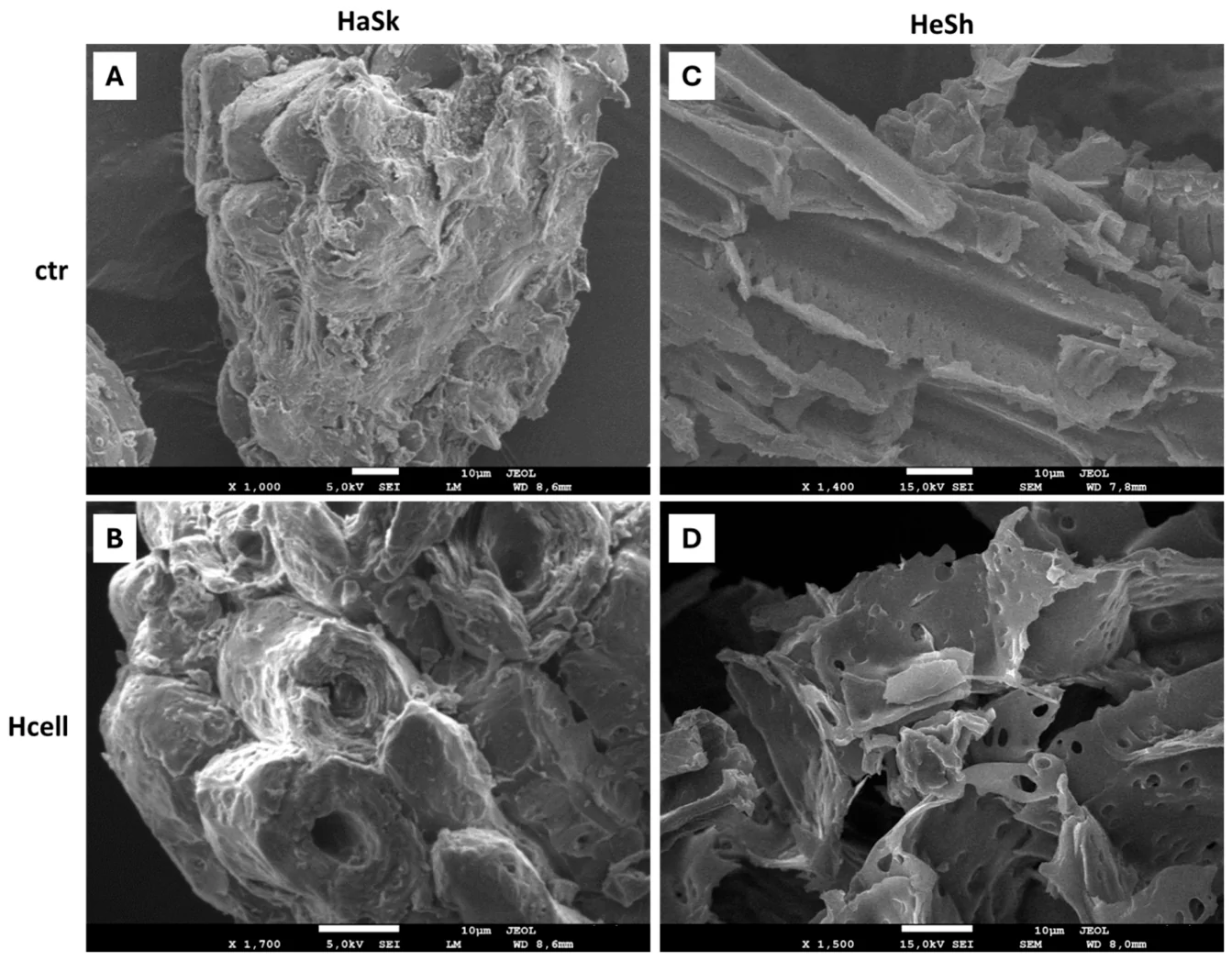
SEM images of hazelnut skins (HaSk) and hemp shives (HeSh) solid residues obtained after 3 h of treatment without (ctr, (**A**,**C**)) and with hemicellulase (Hcell, (**B**,**D**)).

Scanning electron microscopy (SEM) revealed evident morphological modifications in both hazelnut skins (HaSk) and hemp shives (HeSh) following enzymatic treatment with hemicellulase (Figure 3). Overall, the observed structural changes are consistent with the progressive disruption of the lignocellulosic matrix inferred from the release of soluble carbohydrates described in Section 3.2.

Untreated HaSk (Figure 3A) exhibited a compact and heterogeneous morphology characterized by tightly packed lamellar structures and a rough, irregular surface. Following enzymatic hydrolysis (Figure 3B), the biomass became markedly less compact, displaying a more open architecture with the appearance of cavities and partial separation of structural layers. These morphological modifications are indicative of a progressive loosening of the lignocellulosic network, potentially increasing the accessibility of structural polysaccharides to enzymatic attack.

A similar trend was observed for HeSh. Untreated samples (Figure 3C) displayed elongated fibrous elements with relatively compact and continuous cell-wall structures. In contrast, hemicellulase-treated HeSh (Figure 3D) exhibited evident surface erosion, partial delamination of the fibres, and the appearance of numerous pores and fractures, indicating extensive modification of the cell-wall architecture. Compared with HaSk, HeSh appeared to undergo a more pronounced fragmentation of the fibrous structure, likely reflecting the different organization and composition of its lignocellulosic components [20].

These observations are consistent with previous SEM investigations of hemp biomass subjected to pretreatment and enzymatic hydrolysis. Abraham et al. [40] reported the formation of micropores and fragmentation of the biomass structure following enzymatic hydrolysis, highlighting the structural modifications associated with changes in the accessibility of lignocellulosic components. Similarly, Li et al. [41] observed fibre erosion, splitting and separation of structural layers during enzymatic hydrolysis of lignocellulosic fibres. Although differences in biomass composition and treatment conditions prevent direct quantitative comparisons, these studies support the interpretation that the pores, fractures and partial delamination observed in hemicellulase-treated HeSh reflect structural disruption of the biomass.

Therefore, the morphological changes observed in both biomasses support the progressive deconstruction of the lignocellulosic matrix during enzymatic hydrolysis. The increased porosity and disruption of structural integrity are expected to facilitate enzyme penetration into the biomass, thereby enhancing the accessibility of structural polysaccharides and promoting their hydrolysis. These observations are in agreement with the progressive release of soluble carbohydrates discussed in Section 3.2 and confirm the effectiveness of hemicellulase in modifying the structural organization of both lignocellulosic matrices. Similar morphological alterations have been reported following enzymatic treatment of lignocellulosic biomasses, where progressive matrix deconstruction increased substrate accessibility and facilitated carbohydrate recovery [33].

SEM analysis revealed differences in the extent of the morphological modifications induced by the enzymatic treatments. Hemicellulase treatment resulted in the most evident structural changes, whereas the effects observed following cellulase, pectinase, and xylanase treatments were less pronounced under the experimental conditions investigated. Representative SEM images of the different enzymatic treatments are provided in the Supplementary Materials (Figure S3).

This limited effect may be attributed to the restricted accessibility of their specific substrates within the highly organized lignocellulosic network, where cellulose, hemicellulose and lignin form a complex three-dimensional architecture that limits enzyme penetration. Consequently, these enzymes may have induced localized structural alterations that were insufficient to produce evident changes detectable by SEM. Nevertheless, such modifications may still have contributed to the release of soluble compounds, as discussed in the following sections. In contrast, the more pronounced structural disruption observed after hemicellulase treatment is likely related to the complexity of commercial hemicellulase preparations, which generally comprise several accessory glycoside hydrolases acting synergistically on different domains of the hemicellulosic network. This coordinated enzymatic action promotes a more extensive deconstruction of the lignocellulosic matrix than that achievable by individual carbohydrolases.

### 3.4. Structural Characterization by ATR-FTIR Spectroscopy

**Figure 4:**
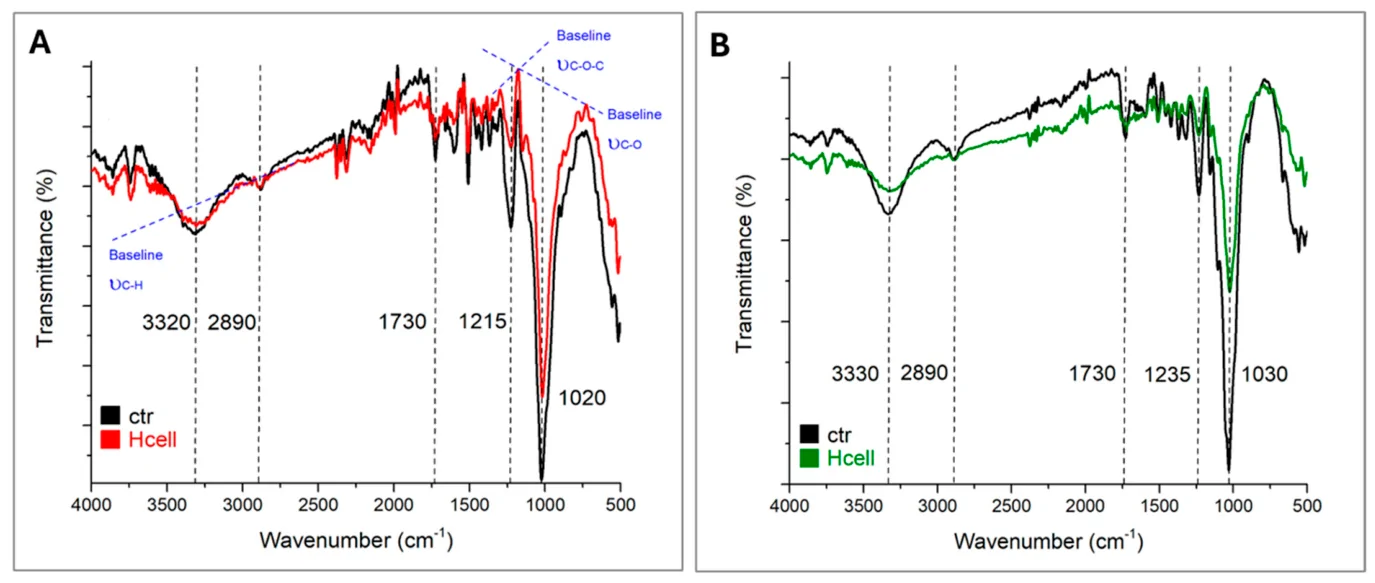
ATR-FTIR of hazelnut skins (HaSk, (**A**)) and hemp shives (HeSh, (**B**)) solid residues obtained after 3 h of treatment without (ctr) and with hemicellulase (Hcell).

ATR-FTIR spectroscopy was performed to investigate the structural modifications induced by enzymatic hydrolysis in hazelnut skins (HaSk) and hemp shives (HeSh). The spectra of untreated and hemicellulase-treated samples, normalized to the C–H stretching band, are compared in Figure 4. This band was selected as an internal reference because the C–H stretching vibrations are not expected to be significantly affected by the enzymatic hydrolysis of hemicellulose.

In general, both biomasses retained the characteristic spectral profile of lignocellulosic materials following enzymatic treatment, indicating that hemicellulase did not extensively modify the overall chemical fingerprint of the matrices.

Nevertheless, distinct variations in the intensity of selected absorption bands were observed, revealing localized structural changes within the polysaccharide network that are consistent with the morphological alterations evidenced by SEM analysis. The assignment of the principal absorption bands was based on previous studies on lignocellulosic materials [42,43].

In both biomasses, the broad absorption band centered at approximately 3330–3320 cm^−1^, assigned to the stretching vibration of hydrogen-bonded hydroxyl groups in structural polysaccharides, exhibited a lower intensity after enzymatic treatment. Changes in this region may reflect modifications in the hydrogen-bonding environment of the polysaccharide matrix following enzymatic treatment associated with the rearrangement of cellulose and hemicellulose chains [44]. The band around 2897 cm^−1^ is assigned to aliphatic C–H stretching vibrations of aliphatic groups and was used as an internal reference for the relative intensity analysis; in fact, minor variations were observed around 2897 cm^−1^, indicating that the hydrocarbon backbone of the lignocellulosic matrix remained substantially preserved [7].

Other relevant spectroscopic modifications were detected in the carbohydrate fingerprint region. In HaSk, the reduced intensity of the absorption band at approximately 1730 cm^−1^, commonly attributed to carbonyl stretching vibrations of acetyl and ester groups in hemicelluloses [42,43], provides evidence of the selective action of hemicellulase on hemicellulosic components.

However, additional changes were observed in the carbohydrate fingerprint region between 1250 and 1000 cm^−1^, which includes bands associated with C–O and C–O–C stretching vibrations of structural polysaccharides [42,45]. To compare the samples before and after enzymatic treatment, the relative intensities of the selected C–O and asymmetric C–O–C stretching bands were expressed as ratios to the C–H stretching band intensity, using the corresponding baselines shown in Figure 4. In HaSk, the C–O/C–H and C–O–C/C–H ratios increased by approximately 1.3-fold and 1.1-fold, respectively, after enzymatic treatment. These changes indicate alterations in the relative spectral contributions of the selected polysaccharide-associated bands and are consistent with modifications of the carbohydrate matrix following hemicellulase treatment [42,45].

A similar trend was observed in HeSh, where the relative intensity ratios increased by approximately 2.2-fold for C–O/C–H and 1.7-fold for C–O–C/C–H and the spectroscopic modifications were more pronounced than those detected in HaSk. In particular, these larger changes suggest a more pronounced alteration in the relative spectral contributions of the selected bands in HeSh than in HaSk, indicating a more extensive hydrolysis and rearrangement of the polysaccharide network following enzymatic treatment. However, these ratios should not be interpreted as direct quantitative measurements of glycosidic bond cleavage. Rather, they provide complementary evidence of changes in the polysaccharide matrix that can be considered alongside the morphological modifications observed by SEM and the release of soluble carbohydrates reported in Section 3.2. These findings are consistent with the higher susceptibility of hemp shives to enzymatic deconstruction, as also suggested by the more evident morphological modifications observed by SEM and by the substantial release of soluble carbohydrates following hydrolysis.

Overall, ATR-FTIR analysis provides complementary information on the structural changes associated with hemicellulase treatment. The observed changes in the relative intensities of selected bands, together with the SEM observations and soluble carbohydrate release, support the occurrence of modifications in the lignocellulosic matrices. Taken together, these findings suggest that hemicellulase progressively increases the accessibility of structural polysaccharides promoting their enzymatic deconstruction under the conditions investigated, while the main spectral features characteristic of the lignocellulosic materials remained detectable. These results, therefore, support its application as a biocatalytic approach, under relatively mild reaction conditions, for the valorisation of agro-industrial residues.

### 3.5. Functional Properties of the Enzymatically Treated Solid Residues

**Figure 5:**
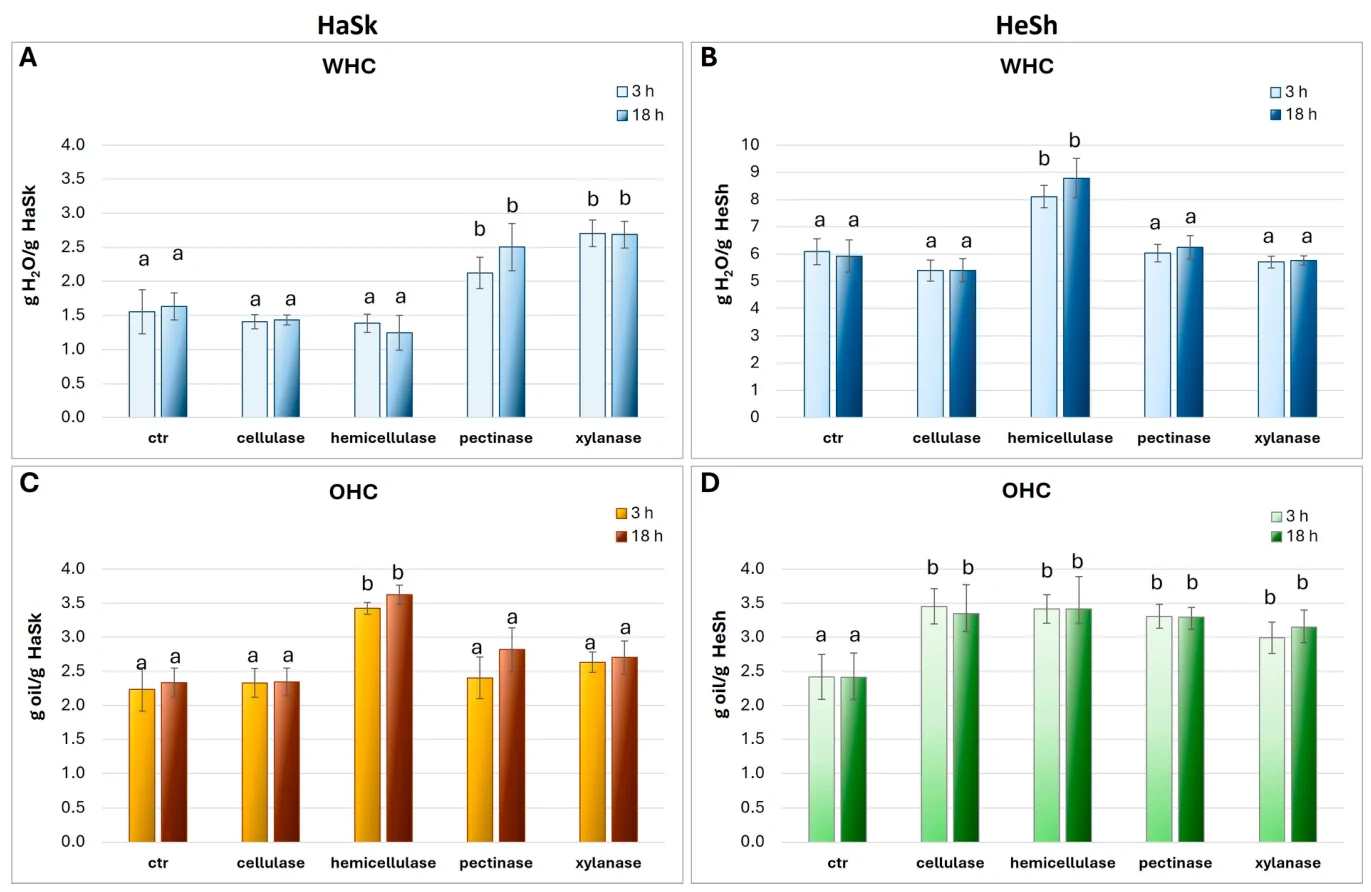
WHC and OHC of hazelnut skins (HaSk, (**A**,**C**)) and hemp shives (HeSh, (**B**,**D**)) solid residues obtained after 3 h and 18 h of enzymatic treatment with different enzymes. Ctr, control treated without enzyme. Data expressed as mean ± SD of three replicates (n = 3). Different letters indicate significant differences (*p* < 0.05).

The structural modifications induced by enzymatic hydrolysis were reflected in the functional properties of the residual solid fractions (Figure 5). Water- and oil-holding capacities are strongly influenced by the chemical composition, porosity and supramolecular organization of lignocellulosic materials. Consequently, enzymatic remodeling of the polysaccharide network can markedly modify the interaction of biomass with water and lipids, although the magnitude and direction of these changes largely depend on the structural characteristics of the substrate and on the enzymatic treatment applied [46,47].

Hazelnut skins exhibited distinct responses in terms of hydration and oil retention. Water-holding capacity was significantly higher in samples treated with pectinase and xylanase than in the control, cellulase- and hemicellulase-treated samples, whereas no significant differences were observed among the latter treatments (Figure 5A). These findings suggest that the selective modification of pectic and hemicellulosic domains may increase matrix hydration by improving the accessibility of hydrophilic sites while preserving the structural integrity of the fibre network.

The variable response of HaSk is consistent with the contrasting effects reported for enzymatically modified dietary fibres. Wen et al. [46] reported a decrease in both WHC and OHC following cellulase- and xylanase-based modification of rice bran dietary fibre, whereas Liu et al. [47] observed increased WHC and OHC after cellulase treatment, which was attributed to increased surface porosity and greater exposure of binding sites. These contrasting findings indicate that enzymatic modification does not produce a univocal effect on hydration properties, but rather depends on the extent and nature of the structural changes induced in the fibre matrix.

A different trend was observed for oil-holding capacity. Among the tested enzymes, only hemicellulase significantly increased OHC compared with the untreated control, whereas cellulase, pectinase and xylanase produced no significant changes (Figure 5C). This selective improvement is likely associated with the partial removal of hemicellulosic components and the consequent exposure of internal surfaces capable of physically entrapping lipids. This result is in agreement with studies showing that enzymatic modification can enhance the oil-binding properties of plant-derived fibres. For example, Shen et al. [48] reported a moderate increase in both WHC and OHC following enzymatic modification of black soybean hull dietary fibre using cellulase and hemicellulase. However, the opposite trend has also been reported for rice bran dietary fibre, highlighting the substrate-dependent nature of the response [46].

The smoother morphology observed by SEM and the structural modifications evidenced by ATR-FTIR support this interpretation, indicating that enzymatic hydrolysis promoted a reorganization of the fibre architecture that altered its functional behaviour. Similar relationships between microstructural rearrangement and changes in hydration and oil-binding properties have been reported following the physical and enzymatic modification of dietary fibres [47,49].

In contrast, hemp shives responded differently to enzymatic treatment. Water-holding capacity remained comparable to the control after cellulase, pectinase and xylanase treatments, whereas hemicellulase significantly increased water retention (Figure 5B). This behaviour is consistent with the more pronounced structural modifications observed by SEM and ATR-FTIR, suggesting that the enzymatic deconstruction of the hemicellulosic network generated a more accessible and porous architecture capable of retaining larger amounts of water. This finding is consistent with the increased hydration properties reported for enzymatically modified plant fibres when hydrolysis promotes the exposure of internal binding sites and the formation of a more accessible matrix [47,48].

Unlike WHC, oil-holding capacity increased significantly after all enzymatic treatments, with no statistical differences among cellulase, hemicellulase, pectinase and xylanase (Figure 5D). These results indicate that even moderate modifications of the lignocellulosic architecture were sufficient to enhance lipid retention in hemp shives, irrespective of the specific carbohydrolase employed. The improvement in OHC is likely related to changes in surface morphology and to the formation of additional physical entrapment sites within the partially disassembled matrix, as previously reported for enzymatically modified dietary fibres.

Interestingly, only minor differences were observed between 3 h and 18 h of treatment for both biomasses, indicating that the structural modifications responsible for changes in hydration and oil retention occurred predominantly during the early stages of enzymatic hydrolysis. This behaviour is consistent with the rapid release of reducing sugars discussed in Section 3.2 and further supports the hypothesis that carbohydrolases rapidly modify the most accessible regions of the lignocellulosic network.

Taken together, these findings demonstrate that enzymatic hydrolysis not only promotes the recovery of soluble compounds but also tailors the functional properties of the residual solid fraction, expanding the opportunities for the integrated valorisation of agro-industrial lignocellulosic residues within a biorefinery framework.

### 3.6. Total Phenolic Content and Antioxidant Activity

**Figure 6:**
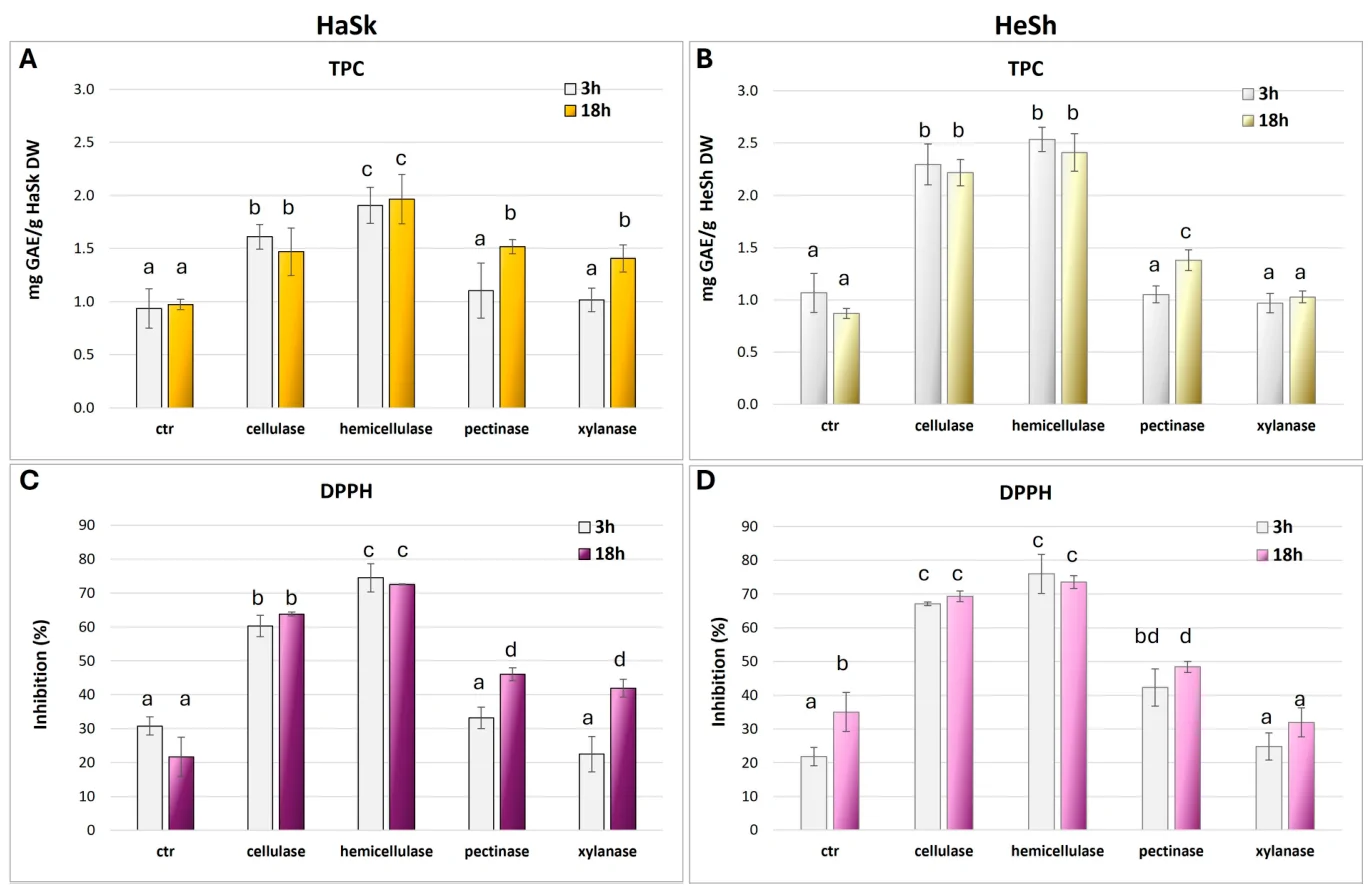
TPC and DPPH antioxidant capacity of the hydrolyzed fractions of hazelnut skins (HaSk, (**A**,**C**)) and hemp shives (HeSh, (**B**,**D**)) after 3 h and 18 h of enzymatic treatment with different enzymes. ctr, control without enzymes. Data expressed as mean ± SD of three replicates (n = 3). Different letters indicate significant differences (*p* < 0.05).

The progressive disruption of the lignocellulosic architecture, demonstrated by sugar release (Section 3.2), SEM observations (Section 3.3) and ATR-FTIR analysis (Section 3.4), was accompanied by an enhanced recovery of phenolic compounds in the liquid fraction (Figure 6). This finding indicates that enzymatic hydrolysis not only promoted the depolymerization of structural polysaccharides but also facilitated the release of bioactive molecules previously entrapped within or associated with the cell-wall matrix.

The effects of enzymatic treatment on total phenolic content (TPC) closely paralleled those observed for antioxidant activity, suggesting a strong relationship between phenolic recovery and the radical scavenging capacity of the hydrolysates. Although the magnitude of the response differed between hazelnut skins and hemp shives, hemicellulase consistently produced the greatest increase in both TPC and antioxidant activity, confirming the effectiveness of this enzymatic preparation in disrupting lignin–carbohydrate complexes and promoting the extraction of bound phytochemicals.

Plant phenolics occur in different forms, including free molecules, soluble conjugates and insoluble-bound compounds esterified or etherified to structural polysaccharides and lignin within the cell wall. Consequently, a considerable proportion of these bioactive molecules remains inaccessible unless the lignocellulosic network is disrupted. The enzymatic degradation of hemicellulose and other matrix polysaccharides increases cell-wall permeability and weakens the interactions between carbohydrates and phenolic compounds, facilitating their transfer into the aqueous phase [15,50]. The superior performance of hemicellulase observed in the present study is therefore consistent with its multienzymatic composition, which includes xylanases, mannanases and accessory carbohydrolases capable of hydrolysing highly branched hemicellulosic domains that frequently constitute the structural framework retaining bound phenolics.

The close correspondence between TPC and DPPH values further supports the hypothesis that the increased antioxidant activity primarily resulted from the enhanced release of phenolic compounds rather than from the production of low-molecular-weight carbohydrates during hydrolysis. Similar relationships between enzymatic disruption of lignocellulosic matrices, increased phenolic recovery and improved antioxidant capacity have been reported for several plant-derived by-products, confirming enzyme-assisted extraction as an effective and environmentally friendly strategy for recovering bioactive compounds from agro-industrial residues [15,51,52].

Altogether, these findings demonstrate that enzymatic hydrolysis simultaneously generated two value-added streams: a liquid fraction enriched in soluble sugars and antioxidant phenolic compounds, and a structurally modified solid fraction exhibiting tailored functional properties. This integrated valorisation approach fully aligns with the biorefinery concept and highlights the potential of enzyme-assisted processing as a suitable strategy for the comprehensive exploitation of lignocellulosic agro-industrial residues.

## 4. Conclusions

This study demonstrated the potential of enzyme-assisted processing as a mild approach for the valorisation of hazelnut skins (HaSk) and hemp shives (HeSh), highlighting the influence of both substrate composition and enzyme type on the response of lignocellulosic matrices. The enzymatic treatments induced different effects on the two materials, confirming that the effectiveness of a given enzyme is closely related to the structural and compositional characteristics of the substrate. Among the enzymes investigated, hemicellulase produced the most pronounced overall effects, particularly in terms of sugar release and modification of the solid matrices.

The structural changes observed by SEM, together with the changes in the functional properties of the treated residues, indicated that enzymatic action altered the organization and accessibility of the lignocellulosic matrix. The effects on water- and oil-holding capacities were substrate- and enzyme-dependent: HaSk showed significant changes in WHC depending on the enzyme applied, whereas HeSh maintained or increased its hydration properties and exhibited an increase in OHC following hemicellulase treatment. These findings indicate that enzymatic processing can modify the functional characteristics of lignocellulosic residues without necessarily resulting in the same response across different substrates.

The analysis of the liquid fractions further demonstrated an increase in total phenolic content and antioxidant activity following enzymatic treatment, suggesting that enzymatic disruption of the lignocellulosic matrix promoted the release of phenolic compounds into the aqueous phase. Although the present study did not include the identification and quantification of individual phenolic compounds, these results provide evidence of the potential of enzymatic treatment to improve the recovery of phenolic-containing fractions from lignocellulosic residues.

## Figures and Tables

**Table 1:** Physicochemical composition of hazelnut skins (HaSk) and hemp shives (HeSh), expressed as percentage of dry weight.

Component	HaSk	HeSh
Ash	2.59 ± 0.44	4.46 ± 1.20
Proteins	3.66 ± 0.19	3.40 ± 0.41
Hexane Extractives	4.22 ± 0.26	2.38 ± 0.15
Cellulose	45.15 ± 0.13	37.11 ± 2.14
Hemicellulose	14.85 ± 0.65	25.12 ± 2.12
Lignin	29.58 ± 4.07	27.59 ± 3.15

## Data Availability

The original contributions presented in this study are included in the article/supplementary material. Further inquiries can be directed to the corresponding author.

## Supplementary Materials

The following supporting information can be downloaded at https://www.mdpi.com/article/10.3390/ma19214589/s1. Figure S1. Particle size distribution of hazelnut skin (HaSk) and hemp shives (HeSh) raw powders (control, ctr) and micronized powders obtained using a hammer mill (HM). Figure S2. Total sugars released in the supernatant after pre-washing of hazelnut skin (HaSk) and hemp shives (HeSh) powders with ethanol, acetone, water at room temperature under stirring for 10 min, and water after incubation in a water bath at 100 °C for 10 min (Water/100 °C). GE, glucose equivalents. Data expressed as mean ± SD of three replicates (n = 3). Figure S3. SEM images of hazelnut skins (HaSk) and hemp shives (HeSh) solid residues obtained after 3 h of treatment with cellulase (Cell, A and D), pectinase (Pec, B and E) and xylanase (Xyl, C and F).

## Abbreviations

The following abbreviations are used in this manuscript:

ATR-FTIR	Attenuated Total Reflectance–Fourier Transformed Infrared Spectroscopy
ctr	control
DPPH	2,2-diphenyl-1-picrylhydrazyl
d.w.	dry weight
GE	Glucose Equivalents
GAE	Gallic Acid Equivalents
HaSk	Hazelnut Skin
HeSh	Hemp Shives
Hcell	Hemicellulase
SEM	Scanning Electron Microscopy
TPC	Total Phenolic Content
