# Supplementary material for: Enzymatic Valorisation of Hazelnut Skin and Hemp Shives By-Products
Source: Materials (Basel). 2026 Oct 29;19(21):4589. doi: 10.3390/ma19214589 (PMC13764906; doi:10.3390/ma19214589)
Supplement: Supplementary file 1 [file materials-19-04589-s001.zip › materials-4529398-supplementary.pdf]

materials

MDPI

Supplementary Materials

Enzymatic valorisation of hazelnut skin and hemp shives by-products

Ester De Martino [1], Angela Sorrentino [2,*], Alfredo Sánchez [3], Reynaldo Villalonga [3], Aniello Falciano [2], Carmine Buonaiuto [2] and Prospero Di Pierro [1,2]

[1] Department of Agricultural Sciences, University of Naples Federico II, Piazza Carlo di Borbone 1, 80055, Portici, Naples, Italy
[2] Centre for Innovation and Development in Food Industry (CAISIAL), University of Naples Federico II, Via Università 133, 80055, Portici, Naples, Italy
[3] Nanosensors and Nanomachines Group, Department of Analytical Chemistry, Faculty of Chemistry, Complutense University of Madrid, Plaza de las Ciencias 2, Ciudad Universitaria, 28040 Madrid, Spain
* Correspondence: angela.sorrentino@unina.it

Pretreatment of lignocellulosic biomass

From an industrial perspective, pretreatment is an essential step for fractionating, utilizing, and transforming lignocellulosic biomass into fuels, polymers, chemical feedstocks, and other value-added materials. Physical pretreatments generally involve grinding or milling to reduce particle size, whereas chemical approaches include processes such as organosolv treatment (Tu and Hallett, 2019). In this research project, both physical and chemical pretreatments were applied. In particular, micronisation was performed to reduce the particle size of the biomass and consequently increase the accessibility of the polymeric matrix to enzymatic action. The processed materials were then subsequently subjected to a cleaning procedure using different solvents, including ethanol, acetone and water, the latter tested under both ambient and boiling conditions.

S1. Micronisation of raw material

Micronisation was carried out using a laboratory hammer mill (LM 3100, Perten) equipped with a 0.5 mm cut-off grid, in order to reduce the particle size of the biomass and enhance polymer matrix accessibility to the enzymes.

The size distribution of hammer-milled HaSk and HeSh samples was determined by the wet dispersion laser light scattering method (Hydro 2000S) with a particle size analyzer (Mastersizer 3000, Malvern, Instruments, Worcestershire, UK). Five measurements from three replicates were used for the analyses. The analyses were performed on the samples dispersed in deionized water until a darkening point of around 5% was achieved in the diffractometer cell. In each measurement, the stirring speed was set at 2000 rpm and an ultrasonic treatment was applied for 15 sec.

Particle size distribution analysis confirmed the effectiveness of the micronization process in reducing the particle size of both hazelnut skins and hemp shives (Figure S1). For hazelnut skins, micronization reduced D10, D50 and D90 from 25.3 ± 5.5, 166 ± 61 and 1710 ± 164 μm in the untreated material to 20.0 ± 6.4, 115 ± 77 and 583 ± 58.9 μm, respectively. The most pronounced reduction was observed for D90, indicating a marked decrease in the coarse particle fraction. Similarly, hemp shives showed a substantial reduction in particle size following hammer milling, with D10, D50 and D90 decreasing from 98.3 ± 8.9, 310.2 ± 28.0 and 1117 ± 100.2 μm to 13.0 ± 9.4, 121 ± 53 and 506 ± 80.9 μm, respectively.

Overall, micronisation markedly shifted the particle size distributions towards smaller dimensions, particularly affecting the median and coarse fractions. This reduction in particle size is expected to increase the specific surface area and improve the accessibility of the lignocellulosic matrix to enzymatic hydrolysis.

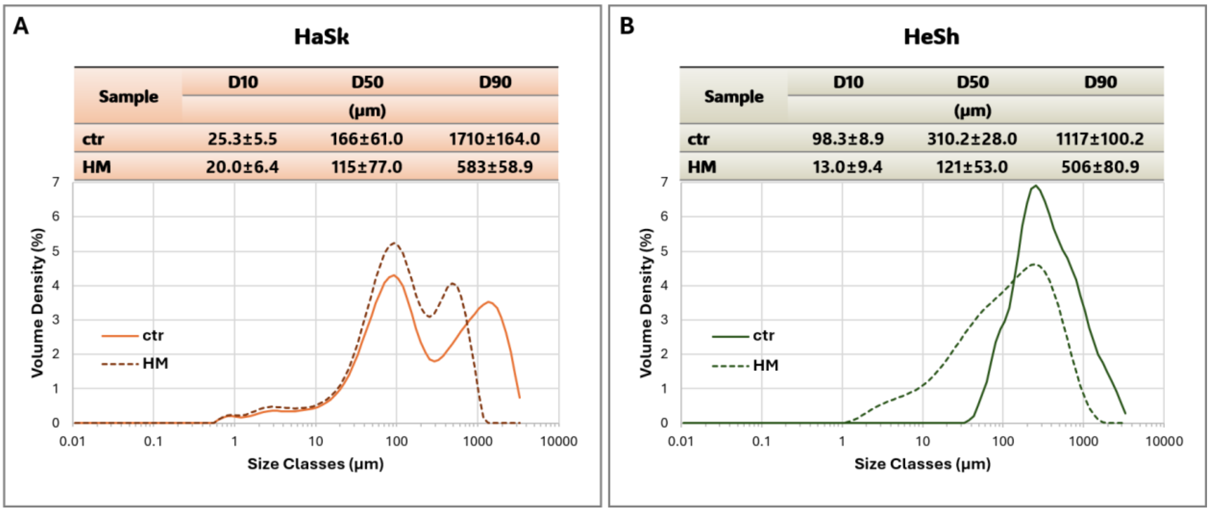

Figure S1. Particle size distribution of hazelnut skin (HaSk, A) and Hemp Shives (HeSh, B) raw powders (control, ctr) and micronized powders obtained using a hammer mill (HM).

S2. Washing treatment

Following micronization, the materials were subjected to a pre-washing step aimed at removing soluble and easily dispersible compounds from the matrix prior to enzymatic treatment. For this purpose, the micronized powders were suspended at a 1:10 solid-to-liquid ratio in different solvents, namely ethanol, acetone or water, and stirred at 800 rpm for 10 min at room temperature. In addition, water treatment was performed under an alternative condition by incubating the suspension at 100°C for 10 min in a water bath. After each treatment, the solid and liquid fractions were separated by centrifugation at 14,000 × g for 10 min, and the supernatant was analysed for total sugar content, expressed as glucose equivalents (GE). As shown in Figure S2, the pre-washing treatment with water at 100°C resulted in the highest release of total sugars compared with the other tested conditions. This finding suggests that hot water was more effective in removing soluble and easily extractable compounds from the biomass. Moreover, the use of water at elevated temperature represents an advantageous pre-treatment from an environmental perspective, as it avoids the use of organic solvents while providing an effective removal of the soluble fraction. Therefore, water treatment at 100°C for 10 min was selected as the most suitable pre-washing condition for subsequent enzymatic hydrolysis.

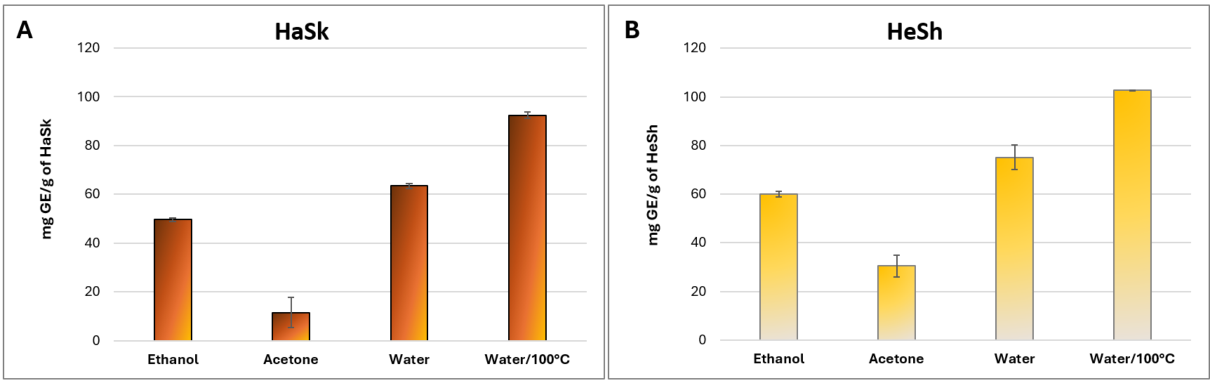

Figure S2. Total sugars released in the supernatant after pre-washing of hazelnut skin (HaSk, A) and hemp shives (HeSh, B) powders with Ethanol, Acetone, Water at room temperature under stirring for 10 min, and with water after incubation in water bath at 100°C for 10 min (Water/100°C). GE, glucose equivalents. Data expressed as mean±SD of three replicates (n=3).

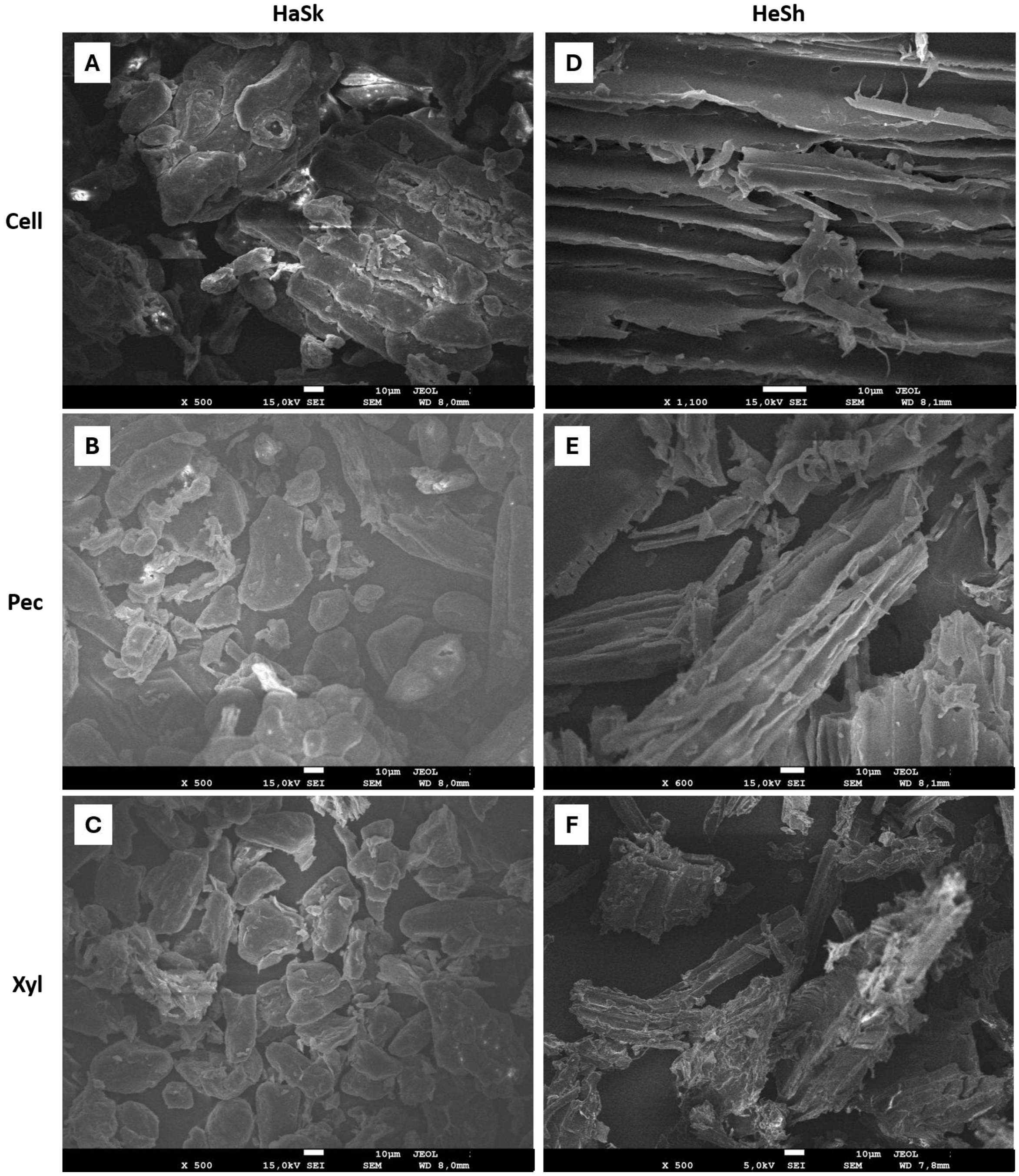

Figure S3. SEM images of hazelnut skins (HaSk) and hemp shives (HeSh) solid residues obtained after 3 h of treatment with cellulase (Cell, A and D), pectinase (Pec, B and E) and xylanase (Xyl, C and F).
